# An ingested metallic wire migrating from stomach to pancreas treated by laparoscopic surgery: A case report

**DOI:** 10.3389/fsurg.2022.927637

**Published:** 2023-01-06

**Authors:** Fulong Hao, Qingbo Feng, Jiaxin Li, Hong Wu

**Affiliations:** ^1^ Department of Liver Surgery and Liver Transplantation Centre, West China Hospital, Sichuan University, Chengdu, China; ^2^ Department of Hepatobiliary Surgery, Suining First People's Hospital, Suining, China; ^3^ Dafang County People's Hospital, Bijie, China

**Keywords:** metallic wire, gastric perforation, pancreas, laparoscopic surgery, case report

## Abstract

**Introduction:**

Foreign bodies inside the pancreas are rare and usually occur after the ingestion of sharp objects such as a fish bone, a sewing needle, or a toothpick. Furthermore, an ingested metallic wire migrating from stomach to pancreas is very rare.

**Case Presentation:**

We report a 36-year-old woman who was admitted to our hospital with “3-day history of dull progressive epigastric pain.” Computed tomography of the abdomen revealed a linear, high-density body between the stomach wall and the pancreas. During the operation, a linear, hard, metallic wire was found in the adhesive tissue between the gastric antrum and the pancreatic body. The operation was uneventful, and the patient recovered well.

**Conclusion:**

The case of a foreign body inside the pancreas caused by a metallic wire is very rare. Radiological examinations play a vital role in the diagnosis of metallic wire ingestion. Metallic wire ingestion can be treated with laparoscopic surgery, both technically and safely.

## Introduction

Majority of foreign bodies that are accidentally ingested pass through the alimentary tract spontaneously within 1 week without complications (1). There is an estimated 1% chance that foreign bodies will penetrate the stomach or small intestine and migrate to other nearby organs, such as the liver and pancreas (2). Once the sharp foreign bodies are inside the pancreas, they will lead to pancreatic abscess, pancreatitis, or pseudoaneurysm, and are misdiagnosed as pancreatic tumors (3–6). Perforation of the metallic wire through the stomach into the pancreas that did not cause fever or pancreatitis is extremely uncommon. We hereby report a rare case of a metallic wire that penetrated through the posterior wall of the gastric antrum, became embedded in the pancreas and was successfully removed laparoscopically.

## Case presentation

**Figure 1:**
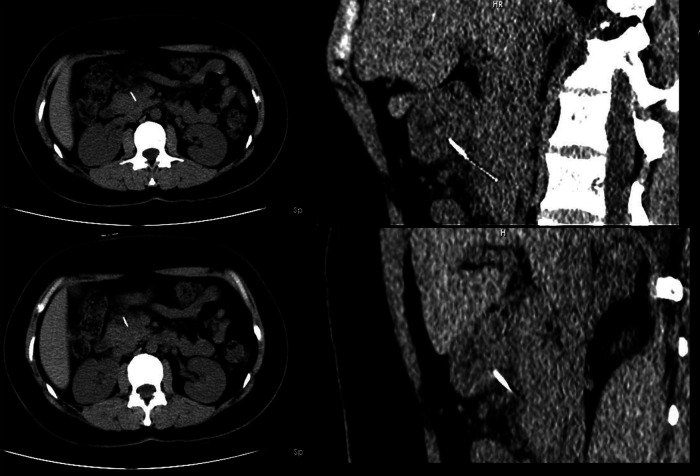
Computed tomography scan showed a hyperdense linear foreign body which penetrated through the posterior wall of the gastric antrum and embedded in the pancreas.

**Figure 2:**
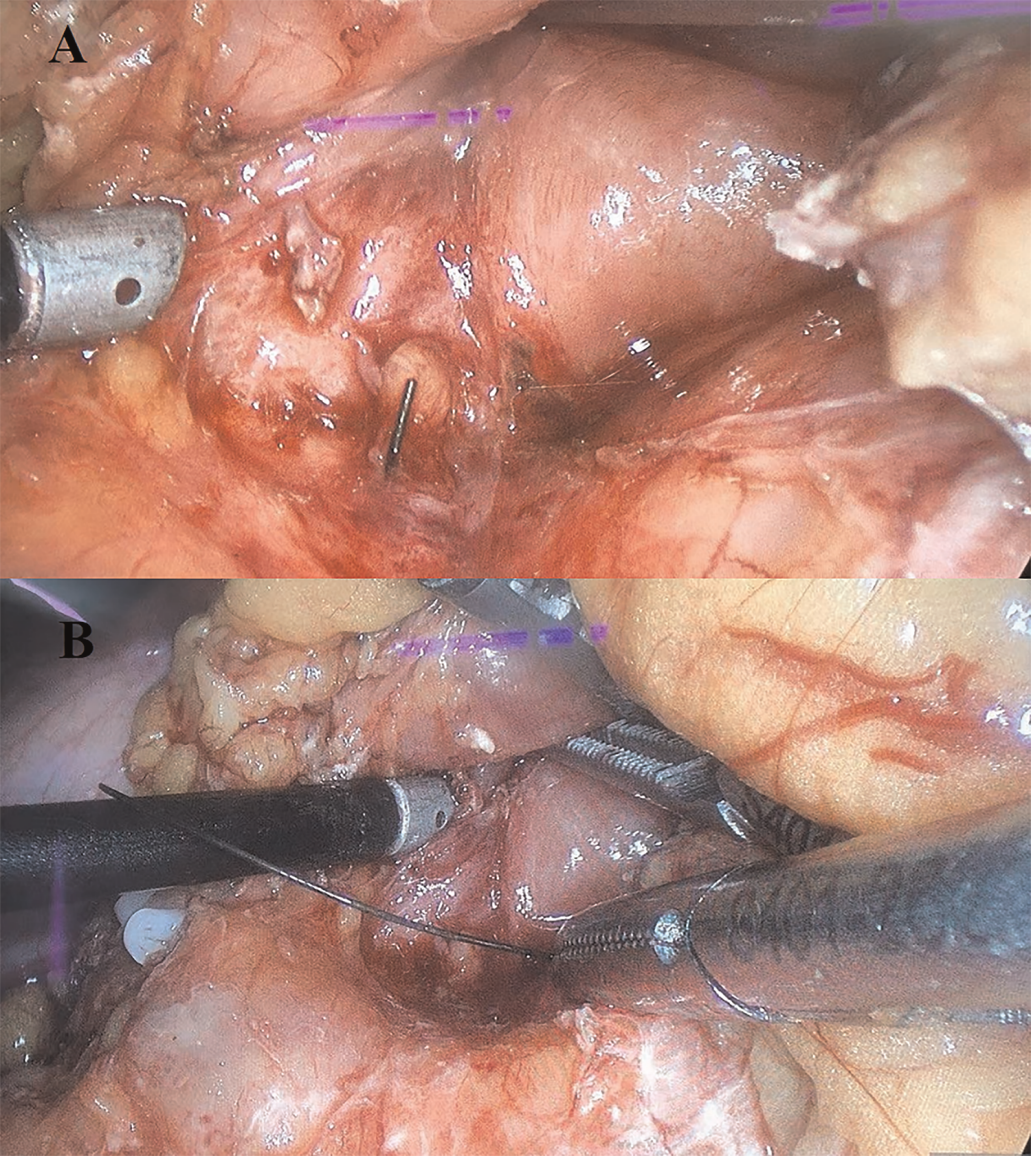
Operative findings. (**A**) A linear, hard, metallic wire was found in the adhesive tissue between the gastric antrum and the pancreatic body. (**B**) Removal of metallic wire.

**Figure 3:**
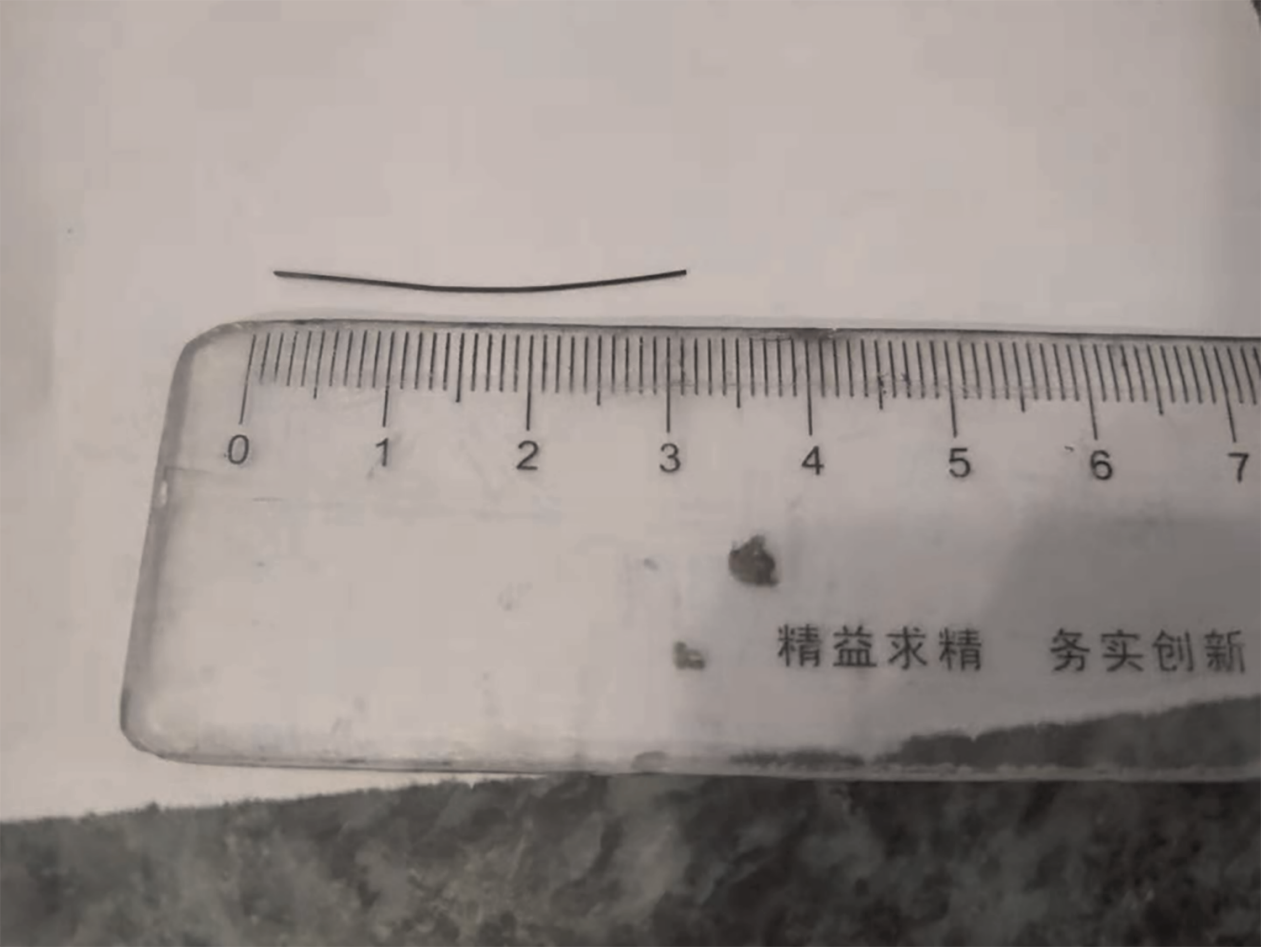
A metallic wire of length 3 cm was extracted from the stomach and pancreas.

Our study was conducted in accordance with the principles of the CASE REPORT (CARE) guidelines (7). A 36-year-old woman presented to our emergency department with epigastric pain for 3 days. Physical examination showed no abdominal tenderness. She had a temperature of 37.1°C, a blood pressure of 113/76 mmHg, a respiratory rate of 18 breaths/min, and a pulse rate of 77 beats/min. No other notable abnormality was found on systemic examination. Initial laboratory investigations were unremarkable except for a markedly elevated white blood cell count of 10.92 × 10^9^/L, with 76.5% neutrophils and slightly elevated levels of C-reactive protein. Abdominal computed tomography (CT) revealed a linear, hyperdense, foreign body that perforated the stomach's posterior wall and was embedded in the pancreas (Figure 1). Abscess formation, free air, and pancreatitis were not observed. Gastroscopy revealed no abnormalities and no obvious perforation. Previous history showed that she was fit and well with no significant medical history. On further questioning, the patient recalled that her family often uses a metallic wire brush to wash pots and pans. This metallic wire may have entered the food in this way, and then she ate it. Diagnostic laparoscopy was performed under general anesthesia. During the operation, the gastrocolic ligament was opened with the help of a laparoscopic instrument. A foreign metallic wire spanning from the posterior of a lesser curvature of the stomach to the head and body of pancreas was visualized (Figure 2). The metallic wire was (3 cm) removed from the abdomen (Figure 3). The defect in the stomach wall was repaired laparoscopically using 4/0 sutures. Symptomatic treatment, including anti-infection, pain relief, and fluid replacement, was routinely applied after surgery. The patient recovered well and was discharged on the 10th day. At 2-months follow-up, the patient had no complications and was doing well.

## Discussion

Foreign body ingestion is a common problem. Most ingested foreign bodies pass spontaneously through the digestive tract, and only 1% of them can perforate or penetrate the gastrointestinal tract and migrate into organs such as the liver and pancreas. When dealing with this condition, it is important for a medical professional to consider the size, sharpness, and shape of the foreign body. When an object is larger than 5 cm or has a pointed shape, the risk of injury increases (8). Up until now, most patients with foreign bodies embedded in the pancreas had a fish bone or a needle (9). To the best of our knowledge, there are only a few cases of an ingested metallic wire that penetrated through the gastrointestinal tract and migrated into the pancreas. Foreign bodies reaching the pancreas can cause abscess, pseudoaneurysm, pancreatitis, and high-mortality-risk complications (3–6). It is important to diagnose gastrointestinal foreign bodies promptly and intervene early to prevent morbidity and mortality (10, 11).

It is often difficult to diagnose these patients since they cannot remember swallowing foreign bodies accidentally. Symptoms can mimic those of epigastric pain or gastritis as well. Abdominal CT examinations can be beneficial in diagnosing and localizing foreign bodies. CT/MRI abdomen detection of foreign bodies is the most common method reported in the literature. Accurate preoperative diagnoses of wire ingestion based on CT is beneficial to the operation of laparoscopic resection. Fortunately, the metallic wire was successfully removed by laparoscopic management in our case. There has been a case where a metal BBQ brush wire was ingested and lodged in the pancreas; in that case, the wire was successfully retrieved with a gastroscopy (12). Based on our experience with the current case, some suggestions should be taken into consideration. Laparoscopic foreign body removal is preferable to open surgical removal when an abdominal foreign body has been diagnosed, especially in patients with a stable, non-acute condition. Laparoscopic surgery has advantages in reducing postoperative pain and wound infection, as well as minimizing surgical stress and taking out the iron wire under the gastroscope. In the present case, there have been a growing number of similar cases treated with laparoscopic surgery over the past few years (4, 11, 13). An ingested metallic wire was successfully removed laparoscopically, and the patient recovered without complications.

In conclusion, we presented a rare case of metallic wire migrating from the stomach to the pancreas. Our case demonstrated that laparoscopy is technically feasible and safe for the treatment of patients with metallic wire ingestion. Imaging examination is helpful for preoperative diagnosis, especially abdominal CT, which is an important tool for both localization and surgical decision making. Most cases can be diagnosed by CT, and patients with foreign body perforation should be treated surgically. Compared with open surgery, laparoscopic minimally invasive surgery provides better diagnosis, less postoperative pain, less wound infection, and a faster recovery.

## Data Availability

The original contributions presented in the study are included in the article/Supplementary Material, further inquiries can be directed to the corresponding author/s.
